# TROL-FNR interaction reveals alternative pathways of electron partitioning in photosynthesis

**DOI:** 10.1038/srep10085

**Published:** 2015-06-04

**Authors:** Lea Vojta, Dejana Carić, Vera Cesar, Jasenka Antunović Dunić, Hrvoje Lepeduš, Marina Kveder, Hrvoje Fulgosi

**Affiliations:** 1 Division of Molecular Biology, Ruđer Bošković Institute, 10000 Zagreb, Croatia; 2 Division of Physical Chemistry, Ruđer Bošković Institute, 10000 Zagreb, Croatia; 3 Department of Biology, JJ Strossmayer University of Osijek, 31000 Osijek, Croatia; 4 Agricultural Institute Osijek, 31000 Osijek, Croatia

## Abstract

In photosynthesis, final electron transfer from ferredoxin to NADP^+^ is accomplished by the flavo enzyme ferredoxin:NADP^+^ oxidoreductase (FNR). FNR is recruited to thylakoid membranes via integral membrane thylakoid rhodanase-like protein TROL. We address the fate of electrons downstream of photosystem I when TROL is absent. We have employed electron paramagnetic resonance (EPR) spectroscopy to study free radical formation and electron partitioning in TROL-depleted chloroplasts. DMPO was used to detect superoxide anion (O_2_^.−^) formation, while the generation of other free radicals was monitored by Tiron. Chloroplasts from *trol* plants pre-acclimated to different light conditions consistently exhibited diminished O_2_^.−^ accumulation. Generation of other radical forms was elevated in *trol* chloroplasts in all tested conditions, except for the plants pre-acclimated to high-light. Remarkably, dark- and growth light-acclimated *trol* chloroplasts were resilient to O_2_^.−^ generation induced by methyl-viologen. We propose that the dynamic binding and release of FNR from TROL can control the flow of photosynthetic electrons prior to activation of the pseudo-cyclic electron transfer pathway.

In vascular plants, photosynthetic electron transport (PET) chain produces reductive power that is utilised by diverse acceptors involved in both chloroplast and cellular metabolism^1^. In the last steps of this process transfer of energy-conserving electrons beyond photosystem I (PSI) is performed by a small iron-sulphur protein ferredoxin (Fd). Fd acts simultaneously as a bottleneck and as a hub which distributes high-energy electrons to a multitude of enzymes, which include nitrite reductase, sulphite reductase, fatty acid desaturase, glutamine-2-oxoglutarate amino transferase (GOGAT), redox complexes such as cytochrome b_6_/f for cyclic electron transport (CET), and thioredoxins^2^. The dominant pathway in chloroplasts is, however, the one that produces NADPH, by the activity of ferredoxin NADP^+^ oxidoreductase (FNR). This is known as the linear electron transfer (LET) pathway^1^. Photosynthetically active FNR (sometimes classified as autotrophic) is a member of the family of soluble monomeric enzymes that contain non-covalently bound FAD (flavin adenine dinucleotide) as the prosthetic group^3^. Autotrophic FNR utilizes two Fds to produce one molecule of NADPH. Other members of the FNR family are involved in various metabolic pathways, such as: nitrogen fixation, isoprenoid biosynthesis, steroid metabolism, xenobiotic detoxification, iron-sulphur cluster biogenesis, and oxidative stress response^4^. In those pathways, FNR however catalyses the opposite reaction, namely the production of reduced Fd^4^, and is thus sometimes classified as heterotrophic FNR. In order to function efficiently Fd/FNR redox chemistry has to be constantly poised^5^. Over-reduction of PET chain can damage protein complexes and result in production of the excess amounts of reactive oxygen species (ROS)^6^. The ROS include free radical forms (O_2_^.−^, superoxide radicals; OH., hydroxyl radical; HO_2_. perhydroxy radical and RO. alkoxy radical) and molecular non-radical forms (H_2_O_2_, hydrogen peroxide and ^1^O_2_, singlet oxygen)^7^. During photosynthesis, PSI and photosystem II (PSII) are the primary sources of ^1^O_2_ and O_2_^.−^ production^6^,^8^. It is widely accepted that in conditions leading to over-reduction, pseudo-cyclic electron transfer enables electrons to flow from water to PSI, with molecular oxygen as alternative electron acceptor^5^. This process, known as Mehler reaction, results in formation of H_2_O_2_ which is generated by the superoxide dismutase (SOD), and converted into water and oxygen by ascorbate peroxidase^9^. Mehler reaction, or water-water cycle, allows functionality of the CET and subsequent synthesis of ATP needed for the Calvin cycle, which finally oxidizes NADPH to re-establish LET^5^. The question remains how is the partitioning of electrons between the various energy-conserving and -dissipating pathways achieved. Thylakoid rhodanase-like protein TROL has been shown to act as *bona fide* membrane attachment point for the FNR^10^,^11^,^12^, although other membrane associations of FNR have been described^13^,^14^,^15^,^16^,^17^,^18^,^19^. Further, the N-terminus of FNR was shown to be important for the FNR recruitment to the membrane and its association with different supramolecular complexes^11^. TROL consists of the centrally positioned rhodanase homology domain which is connected by proline and valine rich swivel with the C-terminal FNR membrane recruitment motif (MRM)^10^,^18^. The MRM of TROL shares high amino acid similarity with a similar domains of the inner envelope translocon protein component Tic62^10^,^18^,^20^. Association of pea Tic62 poly-proline type II helix MRM peptide with the FNR was shown to be pH-dependent^18^. However, Tic62 is a soluble protein and it’s homologues from other plant species lack FNR MRM^18^,^21^. TROL-FNR complexes are located in non-appressed regions of thylakoid membranes, in the vicinity of PSI^10^. TROL knock-out (*trol*) plants have decreased electron transport rate (ETR) and increased non-photochemical quenching (NPQ) in high-light (HL) conditions^10^.

Recently, a new concept for FNR binding to plant thylakoids has been proposed^22^. According to this model, a majority of chloroplast FNR is during periods of darkness bound to thylakoids *via* Tic62 and TROL proteins. In these conditions, surplus FNR is stored at the points of attachment, presumably stabilizing the FNR enzyme during the hours of photosynthetic inactivity. In the periods of changing light conditions, *i.e*. in the morning hours, FNR is released to stroma, where it acts as an efficient NADPH catalyst, allowing efficient LET. However, contrary to this scheme previous studies have reported that NADP+ photoreduction is very inefficient when the enzyme is not bound to the membranes^23^ and that thylakoids devoid of FNR cannot reproduce WT rates of NADPH production^24^. Such scenario cannot be avoided even when high concentrations of soluble FNR are added^24^. Further, in transgenic Arabidopsis plants enriched for FNR at TROL, a more rapid induction of NPQ during light transition and a higher ratio of PSI/PSII excitation were recorded, implicating enhanced CEF^21^.

We have previously proposed a different scheme for the dynamic FNR recruitment to TROL^20^,^25^. We proposed that in normal light conditions TROL bound FNR performs efficient NADP^+^ photoreduction, due to specific FNR recruitment at the vicinity of PSI, which is necessary for the directed transfer of reduced Fd. In the conditions of HL, FNR is released from TROL, probably by the transmembrane signalling involving rhodanase and proline–rich domains. Released FNR can act as efficient ROS scavenger, or Fd can partition electrons to other acceptors.

Here we test our hypothesis and investigate how the deletion of TROL and membrane detachment of FNR from this anchoring site influence partitioning of electrons beyond PSI. We posit that TROL-FNR interaction presents the branching point between electron-conserving and electron-dissipating pathways. We monitored ROS formation and subsequent induction of plant stress-relief responses. We establish that without TROL, light-dependent O_2_^.−^ generation is reduced, while the generation of other ROS is enhanced. Most significantly, O_2_^.−^ formation induced by MV is diminished in chloroplasts lacking TROL, when plants are pre-acclimated to dark and growth light (GL). We propose that other Fd-dependent pathways downstream of PSI and different from the LET become dominant by the dynamic detachment of FNR form TROL, thus suggesting novel mechanism of photosynthesis regulation. Alternatively, efficient scavenging of O_2_^.−^ by FNR-dependent pathways can be envisaged.

## Results

Generation of ROS in isolated chloroplasts from wild-type (WT) and *trol* plants was monitored in light, as well as in dark, when all reaction centres are open. We have used O_2_^.−^ specific spin trap DMPO (5,5-dimethyl-1-pyrroline-N-oxide) and general electron spin trap Tiron (4,5-dihydroxy-1,3-benzenedisulfonic acid) to detect the production of ROS^26^,^27^ in conditions when FNR docking partner TROL is missing from the vicinity of PSI. Moreover, we chose different light acclimation conditions to promote interactions of FNR with different complexes or membrane domains. Since the concentrations of plant antioxidants are important for the interpretation of ROS production that were investigated in EPR experiments, we have quantified the levels of ROS-scavenging enzymes and the extent of lipid peroxidation and protein carbonyls in the WT and *trol* plants acclimated to GL (see Supplementary Fig. S1 and Supplementary Table S1 online). Except for the peroxisomal catalase activity and the levels of H_2_O_2_, all other enzymatic and non-enzymatic components of antioxidative stress response were not significantly different between WT and *trol* plants (see Supplementary Fig. S1 and Supplementary Table S1 online).

### *Trol* chloroplasts accumulate diminished levels of O_2_

**Figure 1:**
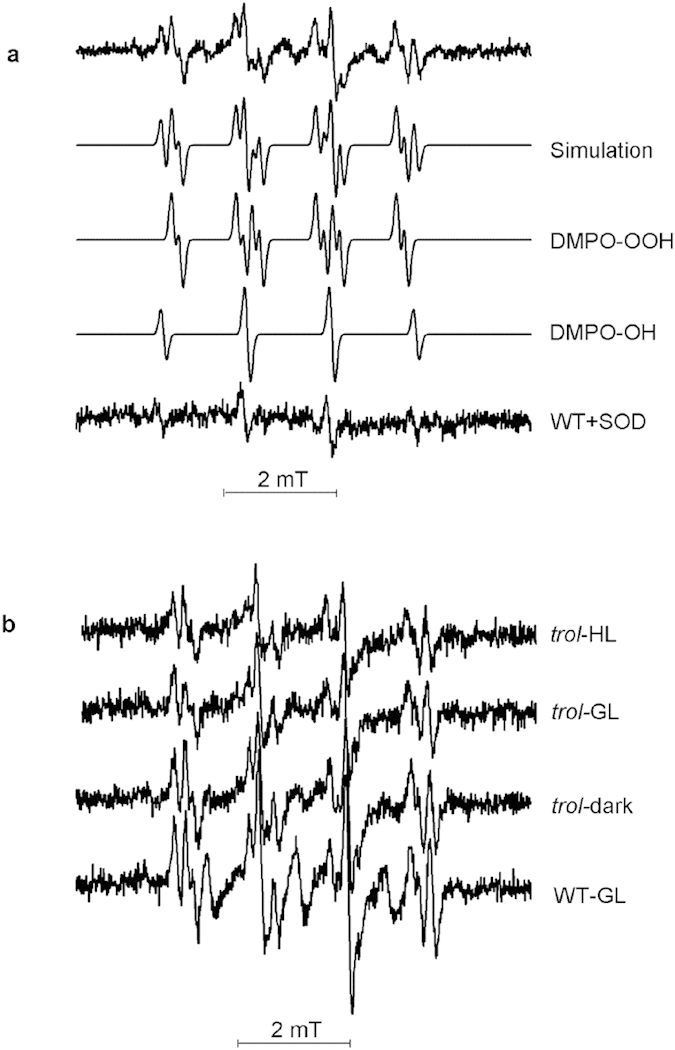
Experimental EPR spectrum. (**a**) The analysis of EPR spectra of DMPO-OOH spin adduct formation in chloroplasts isolated from Arabidopsis WT plants exposed to illumination with photosynthetic light of 100 μmol photons m^−2^ s^−1^ for 30 seconds. The experimental spectrum (WT) was simulated revealing the contribution of DMPO-OOH superoxide spin adduct (75%) and DMPO-OH hydroxyl radical spin adduct (25%). In the presence of SOD the former component is suppressed. (**b**) EPR spectra of spin adducts of chloroplasts isolated from Arabidopsis WT and *trol* plants grown in different light conditions (dark, GL and HL).

**Figure 2:**
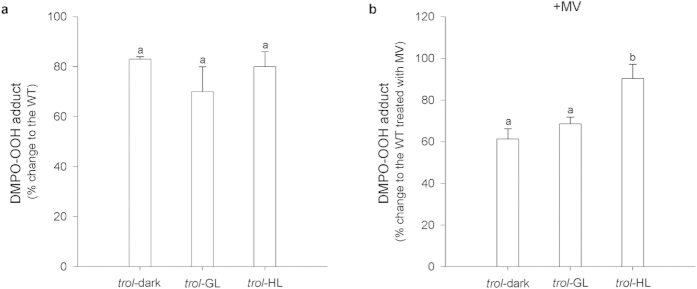
The EPR study of DMPO-OOH spin adduct formation in chloroplasts isolated from Arabidopsis *trol* plants acclimated to different light conditions (dark, GL or HL). Chloroplasts were illuminated with photosynthetic light of 100 μmol photons m^−2^ s^−1^ for 30 seconds. (**a**) The superoxide anion production in mutant expressed in terms of WT plant data exposed to the same experimental conditions and taken as a reference representing 100% radical yield; (**b**) The superoxide anion production from *trol* chloroplasts exposed to MV normalized with respect to the data of WT exposed to MV which are taken as a reference for 100% superoxide radical production. Data (mean ± SE) from three independent measurements were analysed by one-way ANOVA and LSD post-hoc test. Data labelled with the same lower-case letters are not significantly different. Different letters point to the experimental data that are significantly different (p < 0.05).

Illuminated isolated intact Arabidopsis WT chloroplasts doped with DMPO revealed a dominant DMPO-OOH spin adduct formation that, according to the EPR spectral parameters, is typical for the superoxide radical formation (Fig. 1a). The apparent hyperfine splitting constants of DMPO-OOH (a^N^ = 1.42 mT, a^Hβ^ = 1.14 mT, a^Hγ^ = 0.115 mT) are in conformity with the published literature^28^. In the presence of SOD the corresponding EPR signal is suppressed (29, Fig. 1a). As the lifetime of superoxide is short with respect to the time scale of the EPR experiment, the superoxide “end-product” in terms of DMPO-OH hydroxyl radical spin adduct is detected as well (a^N^ = 1.49 mT, a^H^ = 1.47 mT). The analysis of DMPO-OOH spin adduct formation in chloroplasts from *trol* plants acclimated to any of the three light conditions revealed a reduced EPR signal intensity and, thus, decreased free radical production with respect to the WT plants (Fig. 1b). Specifically, when the EPR data measured for *trol* chloroplasts were normalized to the WT data, which are assumed to represent a 100% radical yield, around 20% lower light dependent generation of superoxide anion could be estimated for all three acclimation conditions (Fig. 2a). These findings implicate that in the absence of TROL, photo-generated electrons less efficiently spill over to O_2_, as it could be expected from the present models of the PET^5^, or that the O_2_^.−^ is to some extent scavenged. It is also important to point out that photoreduction of O_2_ by isolated chloroplasts is saturated already at low intensities of light (around 10 μmol photons m^−2^ s^−1^)^18^,^30^ and low concentrations of O_2_. Non-normalised experimental data for the DMPO EPR measurements of WT and *trol* chloroplasts are shown in the Supplementary Table S2.

### Lower amounts of MV-induced O_2_^.−^ can be detected in chloroplasts of dark and growth-light acclimated *trol* plants

To further substantiate these findings and to test previous reports that released FNR acts as an efficient scavenger of O_2_^.−^ anion^31^, we have incubated chloroplasts with methyl-viologen (MV). In illuminated chloroplasts, MV traps almost all electrons from the PSI, specifically from the reduced Fd, at a diffusion-controlled rate^32^, to form its cation radicals, which generate O_2_^.−^ at a rapid rate via auto oxidation. Using EPR spectroscopy, O_2_^.−^ can be detected in terms of the larger DMPO-OOH spin adduct formation in the samples exposed to MV, in comparison to non-exposed ones. In HL conditions the EPR signals did not significantly differ between *trol* and WT chloroplasts. When the experimental data of radical production in chloroplasts non-exposed to MV were taken as referent values representing 100% of O_2_^.−^ yield, it could clearly be noticed that the exposure of WT chloroplasts to MV caused significantly larger EPR signal for plants acclimated to dark (ca. 130%) or GL (ca. 170%) (see Supplementary Fig. S2 online). On the other hand, for WT plants acclimated to HL the yield of O_2_^.−^ was practically not affected by the exposure to MV (see Supplementary Fig. S2 online). In chloroplasts isolated from *trol* plants acclimated to GL, light-induced generation of O_2_^.−^ was drastically increased (ca. 170%) after addition of MV, whereas in chloroplasts isolated from *trol* plants acclimated to dark and HL conditions no significant impact of MV on superoxide anion generation was observed (see Supplementary Fig. S2 online). When the experimental data of radical production in WT chloroplasts treated with MV were taken as referent values representing 100% of O_2_^.−^ yield, it could clearly be noticed that the exposure of *trol* chloroplasts to MV caused significantly smaller EPR signal for plants acclimated to dark (ca. 40% less DMPO-OOH adduct) and to GL (ca. 30% less DMPO-OOH adduct) (Fig. 2b). Observed DMPO-OOH adduct production in chloroplasts of WT and *trol* plants acclimated to HL remains constant despite the addition of MV (Fig. 2b, see Supplementary Fig. S2 online).

### Accumulation of other ROS in *trol* chloroplasts

**Figure 3:**
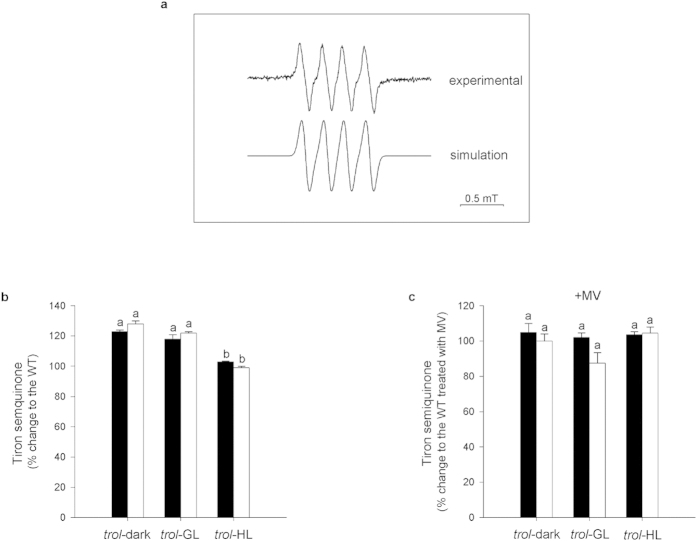
The EPR study of Tiron semiquinone radical formation in chloroplasts isolated from WT and *trol* plants acclimated to different light conditions (dark, GL or HL). Chloroplasts were illuminated with photosynthetic light of 100 μmol photons m^−2^ s^−1^ for 30 seconds (white columns) or kept in dark for the equivalent time period (black columns). (**a**) The experimental and simulated EPR spectra of Tiron semiquinone radical in WT; (**b**) Data are normalized with respect to the EPR signal of the chloroplasts extracted from WT plants assumed to represent 100% radical yield; (**c**) Data from *trol* chloroplasts exposed to MV normalized with respect to the data from WT exposed to MV assumed to represent 100% radical yield. Data (mean ± SE) are analysed by one-way ANOVA and LSD post-hoc test. Data labelled with the same lower-case letters are not significantly different. Different letters point to the experimental data that are significantly different (p < 0.05).

We have then asked whether observed reduction of O_2_^.−^ generation is a part of a more general ROS avoidance response which is dependent on the release of FNR from TROL. To answer this question we have inspected the possibility of other ROS formation, besides the superoxide anion, by using nonspecific electron spin trap Tiron. The appearance of the EPR signal exhibiting electron hyperfine couplings of 0.36 and 0.187 mT from two non-equivalent hydrogens is indicative of Tiron semi-quinone radical (Fig. 3a) pointing to the electron addition sources in general^27^. Since the focus of this study relies on the comparison between the radical productions in mutant versus WT plants, the later experimental data were taken as referent ones and assumed to represent 100% radical yield. In this approach EPR signal intensity from Tiron incorporated in *trol* versus WT chloroplasts was at least 20% larger in either dark- or GL-acclimated plants, in both dark and light EPR experimental setups (Fig. 3b). However, Tiron-detectable ROS in HL-acclimated plants did not significantly differ from the WT (Fig. 3b). Non-normalized experimental data for the WT and *trol* chloroplasts in the presence of Tiron spin trap are shown in the Supplementary Table S2 online. We then performed the experiments in the presence of MV. Contrary to the experiments with DMPO, in the presence of Tiron chloroplasts of both WT and *trol* plants acclimated to any of the three light conditions accumulated significantly more (≥ 50%) Tiron semi-quionone radicals when exposed to MV in comparison to non-exposed, the later assumed to represent the referent 100% radical yield (see Supplementary Fig. S2 online). However, when the EPR signals from WT chloroplasts treated with MV were taken as referent values representing 100% of Tiron semi-quinone yield, it became evident that *trol* chloroplasts in the presence of MV do not generate more Tiron-detectable ROS then the MV treated WT (Fig. 3c). The exception are chloroplasts of GL-acclimated *trol* plants, which generated about 10% less Tiron-detectable ROS when the EPR spectra were recorded under illumination (Fig. 3c), however, statistically non-significantly.

Finally, we have determined the intactness rate of around 75% for both WT and *trol* chloroplasts by the end time point of all described EPR measurements, as assayed by oxygen evolution measurements^33^.

The fact that the generation of these radical species was independent of light exposure in the EPR assays, indicated that generated ROS most likely involve radicals other than the O_2_^.−^ anion.

## Discussion

This work addresses a fundamental question in current photosynthesis research, namely the understanding of the regulation of photosynthetic electron partitioning^21^.

**Figure 4:**
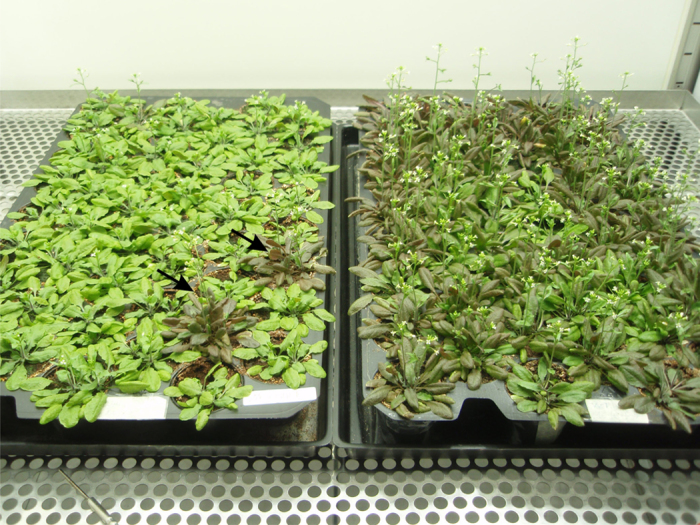
Phenotype of *A. thaliana* plants. Distinctive HL phenotype of *trol* plants (left) vs WT (right) which is evident after 2-day exposure to 800 μmol photons m^−2^ s^−1^. *Trol* plants remain green, while WT accumulates anthocyanins and starts flowering. Both conditions are considered as signs of photooxidative stress. Tray containing *trol* pants was randomly spiked with WT (marked by arrows).

It has been shown that both overexpression of FNR^34^ and its release from thylakoid membranes^31^ increase ROS tolerance in tobacco and wheat. These findings and the distinctive HL phenotype of *trol* plants (Fig. 4) prompted us to investigate stress-relief mechanisms and chloroplast ROS generation in *trol* plants pre-acclimated to different light regimes. To study the generation of ROS and to address the fate of electrons in reactions downstream of PSI, we have employed a highly sensitive electron paramagnetic resonance (EPR) spectroscopy^30^. In chloroplasts, the major site of O_2_^.−^ production is the thylakoid membrane-bound primary electron acceptor of PSI^35^. The generation of O_2_^.−^ may trigger the formation of more reactive ROS like OH., and ^1^O_2_^9^,^30^,^35^. We speculated that the binding of FNR to the TROL is important for partitioning of electrons toward energy-conserving pathway^36^ of NADPH production.

The EPR spin-trapping data on isolated chloroplasts indicate that there are differences in free radical generation or scavenging in WT and *trol* samples. *Trol* chloroplasts generate diminished levels of O_2_^.−^ in all three tested acclimation conditions. It seems that in the absence of TROL photo-generated electrons less efficiently spill over to O_2_. On the other hand, it is possible that in the absence of TROL, PSI photo-generated electrons are not passed over to O_2_ in the first place, but reduced Fd distributes them efficiently to any of the alternative pathways in chloroplasts (see Introduction). Here we have to point out that TROL deficiency does not alter CET^10^. This would imply that the FNR-driven production of NADPH in LET is actually a rate limiting process and that some other Fd-dependent pathway is (are) more efficient electron sink(s). That could explain the need for TROL as an FNR sequestration site in vascular plant thylakoids. Hence, FNR has to be docked in the vicinity of Fd reduction site to prevent Fd from transferring electrons to alternative pathways, although FNR has high affinity for reduced Fd^37^. Finally, it has been shown that Fd-reduced, bound FNR cannot catalyse the photo-reduction of O_2_^38^.

It is generally accepted that during the periods of photosynthesis the role of FNR in the production of NADPH becomes dominant over its diaphorase activity (3 and references therein). In HL, stroma is over-reduced, leading to the activation of FNR diaphorase activity and efficient ROS avoidance (see scheme in 20,25) through so-far unknown mechanism. *Trol* chloroplasts produce approximately 20% less O_2_^.−^ compared to the WT in all pre-acclimation conditions (Fig. 2a). Even more strikingly, in *trol* chloroplasts treated with the redox-cycling agent MV, 30-40% less O_2_^.−^ can be detected, when compared to the MV-treated WT chloroplasts (Fig. 2a). Apparently, chloroplasts of dark- and GL-acclimated *trol* plants distribute electrons in such a way that MV-induced rapid O_2_^.−^ production is efficiently outcompeted, or entirely avoided. Alternatively, in the absence of TROL-FNR interaction, soluble FNR acts as an efficient O_2_^.−^ scavenger, or FNR could be bound to alternative complexes or membrane domains which allows that light-induced generation of O_2_^.−^ is efficiently and rapidly diminished even in the presence of MV. It is possible that in the HL-acclimated plants the mechanisms of O_2_^.−^ scavenging have reached their maximum, irrespective of TROL-FNR interaction.

In the case of the treatments with MV followed by the free radical detection with Tiron, MV consumes almost all electrons from PSI to generate superoxide anion in redox-cycling process. This radical form is subsequently quickly transformed into other radicals. Since *trol* chloroplasts are capable of more efficient scavenging of superoxide anion radicals produced by MV, it is conceivable to assume that the generation of other free radicals is also subsequently lower (compare Supplementary Fig. S2 online, WT-dark to *trol*-dark, and WT-GL to *trol*-GL). Consequently, Tiron can detect less other radical forms. Here, for clarity, it has to be repeated that in the absence of MV *trol* chloroplasts produce 20% more Tiron-detectable radicals than the WT (Fig. 3a).

In conclusion, three scenarios can be envisaged: first, it is possible that scavengers utilized by the free FNR contribute to the generation of other radical forms; second, Fd efficiently passes electrons to alternative pathways when FNR is not sequestered to the TROL, which contributes to over-reduction of other pathways; third, electrons leak to various sinks upstream of the PSI, as the absence of the FNR sequestration site causes slower rates of NADP^+^ reduction and results in over-reduction of LET.

It has been proposed that FNR binding to the membrane recruitment motif of TROL and Tic62 is mediated by changing pH and that this binding is regulated by altering light quantity conditions^11^,^22^. Further, the assembly of different FNR-containing complexes has been implicated in regulation of electron flow^39^,^11^. Also, it has been proposed that the recruitment of FNR to TROL and to the other anchoring proteins is dynamic^10^,^11^. Such alternative binding and release of FNR or its association with different complexes, or electron donors/acceptors could be linked to the regulation and prioritization of different electron transfer pathways^11^,^21^,^22^,^39^. In our models, we suggest that *trol* chloroplasts are not capable of performing the dynamic light- and/or pH-dependent FNR association with the thylakoid membranes^20^,^25^. Finally, Lintala *et al.*^12^ have shown that *tic62 trol* double mutants displays distinct metabolic phenotype, while Goss *et al.*^40^ link FNR amounts and distribution with redox state of cellular glutathione.

To summarize, PSI is capable of reducing O_2_ to O_2_^.−^ 2 and the probability of this occurring increases when the rate of reduction of Fd by PSI exceeds the rate of NADPH utilization by carbon fixing reactions^41^. The maintenance of electron transport under these conditions is particularly important, since the resulting over-acidification of the thylakoid lumen down-regulates excitation of PSII^42^. Present models predict that pseudo-cyclic electron flow, over H_2_O_2_ to water, is activated to restore the redox poise and sustain LET^1^,^5^. Here we demonstrate that the absence of TROL triggers much more efficient O_2_^.−^ scavenging mechanisms. We propose that the dynamic binding and release of FNR from TROL represents novel and efficient mechanism that maintains LET before pseudo-cyclic flow is activated. Furthermore, we hypothesize that the FNR-TROL branch point could be the source element that modulates various downstream networks of plant ROS detoxification.

## Methods

### Plant material and growth conditions

*Arabidopsis thaliana* (L.) ecotype Columbia (Col-0) plants and *At4g01050* knock-out mutant line, *trol*^10^, were grown on soil at 20 °C under 80 μmol photons m^−2^ s^−1^, a 16-h photoperiod and a relative air humidity of 60% (day) and 70% (night). Two days prior to EPR measurements plants were grown either in dark or under 80 μmol photons m^−2^ s^−1^ (GL) or 250 μmol photons m^−2^ s^−1^ (HL). *Trol* line has been thoroughly characterized previously^10^ and has been shown to have molecular and phenotypic characteristics similar to TROL antisense line.

### Chemicals

The spin trap 5,5-dimethyl-1-pyrroline-N-oxide (DMPO), 4,5-dihydroxy-1,3-benzenedisulfonic acid (Tiron) and N,N′-dimethyl-4,4′-bipyridinium dichloride (Methyl viologen dichloride hydrate, MV) were purchased from Sigma Chemical Co. (St. Louis-Aldrich Corp., MO, USA).

### Chloroplast isolation from *A. thaliana*

Intact Arabidopsis chloroplasts were isolated from 3-4 weeks old plants as described by Jurić *et al.*^10^. Final sedimentation was achieved by centrifugation at 1000 xg for 5 min. Sediment was resuspended in buffer containing 330 mM Sorbitol and 20 mM Tris/HCl pH 8.4. Chloroplast concentration was set to 1 mg chlorophyll per 1 ml buffer. Chloroplast intactness and functionality was assessed by oxygen evolution measurements^33^. The value of intactness is given in the Results section.

### EPR measurements

The formation of ROS was measured by EPR spectroscopy using DMPO as a specific superoxide anion spin trap molecule and Tiron as a general spin trap^22^,^23^. Reaction mixture contained chloroplasts equivalent to 50 μg chlorophyll and 10 mM Tiron or 433 mM DMPO whereas in the experiments with MV its respective concentration was 10 mM. Immediately after admission of a specific spin trap, samples were either kept in the dark or illuminated with photosynthetic light of 100 μmol photons m^−2^ s^−1^ for 30 seconds. The experiments were performed in glass capillaries (inner diameter of 1 mm) on an *X*-band Varian E-109 spectrometer applying the following instrumental set-up: microwave power of 20 mW, modulation amplitude of 0.1 mT, modulation frequency of 100 kHz and scan range of 8 mT (DMPO) and 2.5 mT (Tiron). Data were collected using the supplied software^43^ at room temperature and analysed by EasySpin software package^44^. In order to compare the EPR detected radical yield in *trol* versus WT plants, data related to mutant were normalized with respect to the WT data, which were taken as a referent ones and assumed to represent 100% radical yield. Similarly, when the samples were exposed to MV, the results were quantified with respect to the EPR signal of chloroplasts not exposed to MV, which were taken as a referent ones and assumed to represent 100% radical yield.

### Statistics

All statistical analyses were carried out by using IBM SPSS Statistics software, ver. 18 (IBM Corporation, Armonk, New York, USA). Details for the particular analyses are described in the respective Figure and Table legends.

## Additional Information

**How to cite this article**: Vojta, L. *et al*. TROL-FNR interaction reveals alternative pathways of electron partitioning in photosynthesis. *Sci. Rep.* **5**, 10085; doi: 10.1038/srep10085 (2015).

## Supplementary Material

Supplementary Information
