# Supplementary material for: TROL-FNR interaction reveals alternative pathways of electron partitioning in photosynthesis
Source: Sci Rep. 2015 Jun 4;5:10085. doi: 10.1038/srep10085 (PMC4455228; doi:10.1038/srep10085)
Supplement: Supplementary Information [file srep10085-s1.pdf]

TROL-FNR interaction reveals alternative pathways of electron partitioning in photosynthesis

Lea Vojta[a1], Dejana Carić[b1], Vera Cesar[c], Jasenka Antunović Dunić[c], Hrvoje Lepeduš[d], Marina Kveder[b], Hrvoje Fulgosi[a*]

[a]Division of Molecular Biology, Ruđer Bošković Institute, 10000 Zagreb, Croatia

[b]Division of Physical Chemistry, Ruđer Bošković Institue, 10000 Zagreb, Croatia

[c]Department of Biology, JJ Strossmayer University of Osijek, 31000 Osijek, Croatia

[d]Agricultural Institute Osijek, 31000 Osijek, Croatia; Current address: Department of Psychology, Faculty of Humanities and Social Sciences, 31000 Osijek, Croatia

[1]Both authors contributed equally to this work

[*]To whom correspondence may be addressed. E-mail: fulgosi@irb.hr

Corresponding author: Hrvoje Fulgosi
Division of Molecular Biology
Ruđer Bošković Institute
Bijenička cesta 54
HR-10 000 Zagreb, Croatia
Fax: +385 1 4561 177
e-mail: fulgosi@irb.hr

Supplemental Methods:

Measurements of enzymatic and non-enzymatic anti-oxidative components. Activities of CAT, SOD and GR, as well as H_2O_2 and lipid peroxidation levels, ascorbic acid (AA) concentration and protein carbonyls were assayed in leaf extract. Samples were taken from GL conditions (in 16th hour of 80 μmol photons $m^{-2} s^{-1}$), dark (in 8th hour) and HL conditions (after 2 hours of 800 μmol photons $m^{-2} s^{-1}$). For all analyses leaves were grinded into fine powder using liquid nitrogen (with the addition of polyvinyl pyrrolidone (PVP) for CAT, SOD and GR activity). All measurement were done using Specord 40 (Analitic Jena).

Antioxidant enzyme assays. Powdered fresh samples were extracted with 100 mM ice-cold potassium phosphate buffer (pH 7.5) containing 1 mM EDTA for CAT and SOD assays. CAT activity was determined according to Aebi (R1). Two ml reaction mixture contained 50 mM potassium phosphate buffer (pH 7.5), 10 mM H_2O_2 and 20–40 μl of enzyme extract. The decrease of absorbance at 240 nm (caused by decomposition of H_2O_2) was measured. SOD activity was determined according to Giannopolities and Ries (R2). The reaction mixture contained 50 mM potassium phosphate buffer (pH 7.5), 13 mM methionine, 75 μM NBT, 0.1 mM EDTA, 2 μM riboflavin, and enzyme extract. The mixture was illuminated (~120 μmol photons $m^{-2} s^{-1}$) at room temperature for 10 minutes and the absorbance was read at 560 nm. The same one, without illumination, was used as blank. One unit of activity was defined as the amount of enzyme required to cause 50% inhibition of reduction of nitroblue tetrazolium (NBT), compared to the one without enzyme. The extraction for GR activity were done in 100 mM ice cold potassium phosphate buffer (pH 7.0) containing 5 mM sodium ascorbate and 1 mM EDTA. The reaction mixture contained 100 mM potassium phosphate buffer (pH 7.5), 1 mM EDTA, 2 μM GSSG, 2 μM NADPH and enzyme extract. GR activity was assayed by following the decrease of absorbance caused by the oxidation of NADPH at 340 nm, according to Bitensky (R3).

Determination of TBARS, H_2O_2 levels and protein carbonyls. The concentration of thiobarbituric acid-reacting substances (TBARS), as a measure of lipid peroxidation, was monitored as described by Verma and Dubey (R4). Powdered fresh samples were extracted with 1 ml 0.1% (w/v) trichloroacetic acid (TCA). After centrifugation at 6000xg for 5 min, 0.5 ml of the supernatant was added to 1 ml 0.5% (w/v) thiobarbituric acid (TBA) in 20% TCA. The mixture was

heated at 95°C for 30 min and cooled in an ice-bath. After centrifugation (18,000xg, 15 min, 4°C) the absorbance of the supernatant was measured at 532 nm (correction was done by subtracting the absorbance at 600 nm for unspecific turbidity), and the concentration of lipid peroxidation products was expressed as total TBARS in terms of nmol g^{-1} dry weight (extinction coefficient 155 mM^{-1} cm^{-1}). For the determination of H_2O_2 levels powdered leaf tissue was extracted with ice-cold acetone and centrifuged. Titanium sulphate and NH_4OH were added into extracts. The resulting pellet was dissolved with 2 M H_2SO_4 and absorbance was read at 415 nm. The concentration of H_2O_2 was calculated using an extinction coefficient of 1.878 mM^{-1} cm^{-1}, according to Mukherjee and Choudhuri (R5). Powdered samples for determination of carbonyl groups were extracted with 100 mM potassium phosphate buffer (pH 7.5). Homogenates were assayed according to the method of Levine et al. (R6) in which carbonyl groups on protein side were derivatised to 2,4-dinitrophenylhydrazone by reaction with 2,4-dinitrophenylhydrazine (DNPH) dissolved in HCl. The protein contents were determined on HCl-blank pellets and absorbance was read at 280 nm. The content of carbonyl groups was calculated using a bovine serum albumin (BSA) standard curve (BSA is dissolved in urea HCl).

Determination of AA concentration. As a non-enzymatic antioxidant, AA was determined according to Mukherjee and Choudhuri (R5). Powdered fresh samples were extracted in 6% TCA. After centrifugation, 2% DNPH in 50% H_2SO_4 and 10% thiourea in 70% ethanol were added into the supernatant. The mixture was boiled in water bath for 15 min, cooled at RT and centrifuged. Resulting precipitate was dissolved in 80% H_2SO_4 and the absorbance was read at 530 nm. An extinction coefficient of 226.2 $mM^{-1}cm^{-1}$ was used for calculation.

Statistics. All statistical analyses were carried out by using IBM SPSS Statistics software, ver. 18 (IBM Corporation, Armonk, New York, USA). Details for the particular analyses are described in the respective Figure and Table legends.

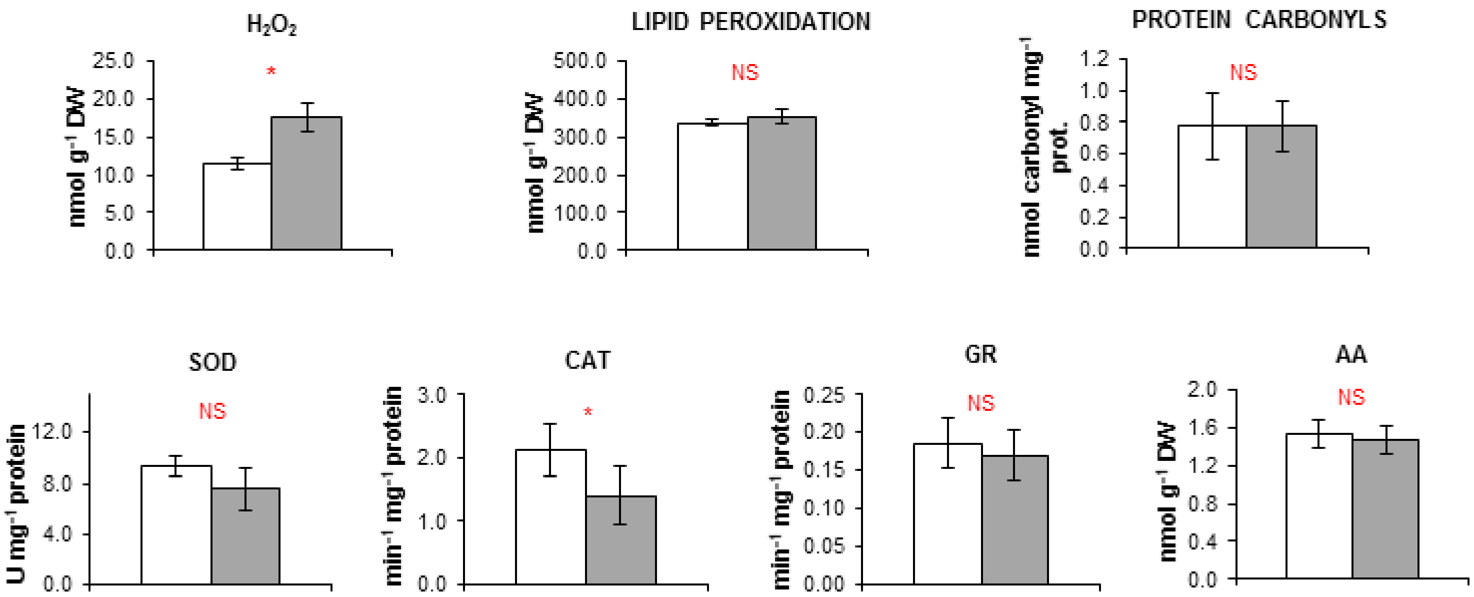

Supplemental Figure S1. Measurements of enzymatic and non-enzymatic components of antioxidative stress response in Arabidopsis WT and *trol* plants grown under growth light. Hydrogen peroxide (H_2O_2); lipid peroxidation level; protein carbonyls; enzyme assays: superoxide dismutase (SOD), catalase (CAT) and glutathione reductase (GR); and ascorbic acid concentration (AA) were detected and quantified in WT (white columns) and *trol* (grey columns) plant leaves after 16 hours constant GL period (80 µmol photons m^{-2} s^{-1}). Each column represents arithmetic mean ± SE. Significant differences between the values ($p<0.01$) are indicated by asterisk. NS depicts non-significant difference between the values. In depth statistical analyses of significances and correlations, as well as N-values are presented in the Supplemental Table S1.

Supplemental Table S1. Mann-Whitney U test of measurements of enzymatic and non-enzymatic components of antioxidative stress response in Arabidopsis WT and *trol* plants grown under the growth light (80 µmol photons m^{-2} s^{-1}). Lipid peroxidation level; hydrogen peroxide (H_2O_2); catalase (CAT); ascorbic acid concentration (AA), superoxide dismutase (SOD); protein carbonyls; and glutathione reductase (GR) were detected and quantified in WT and *trol* plant leaves. All data were analysed by Mann-Whitney U test. Significant differences between the values are indicated by asterisk (significantly higher value, $p<0.01$).

		N	M	SD	Mean rank	Mann-Whitney U (Exact Sig.)
**LP**	**WT**	8	336.37	6.088	5.75	U=10<br>Z=-1.807;<br>p=0.081
	trol	6	352.22	19.160	9.83	
**H_2O_2**	**WT**	8	11.47	0.787	4.50	U=0;<br>Z=-3.24;<br>p=0.000
	trol	7	17.52	1.947	**12.00***	
**CAT**	**WT**	7	2.13	0.404	**10.57***	U=3;<br>Z=-2.747;<br>p=0.004
	trol	7	1.40	0.454	4.43	
**AA**	**WT**	8	1.52	0.142	9.63	U=23;<br>Z=-0.945;<br>p=0.382
	trol	8	1.46	0.149	7.38	
**SOD**	**WT**	6	9.38	0.824	8.50	U=6;<br>Z=-1.922;<br>p=0.065
	trol	6	7.55	1.652	4.50	
**CARBONYLS**	**WT**	8	0.77	0.206	7.44	U=23.5;<br>Z=-0.065;<br>p=0.95
	trol	6	0.77	0.164	7.58	
**GR**	**WT**	6	0.19	0.033	7.33	U=13;<br>Z=-0.801;<br>p=0.485
	trol	6	0.17	0.032	5.67	

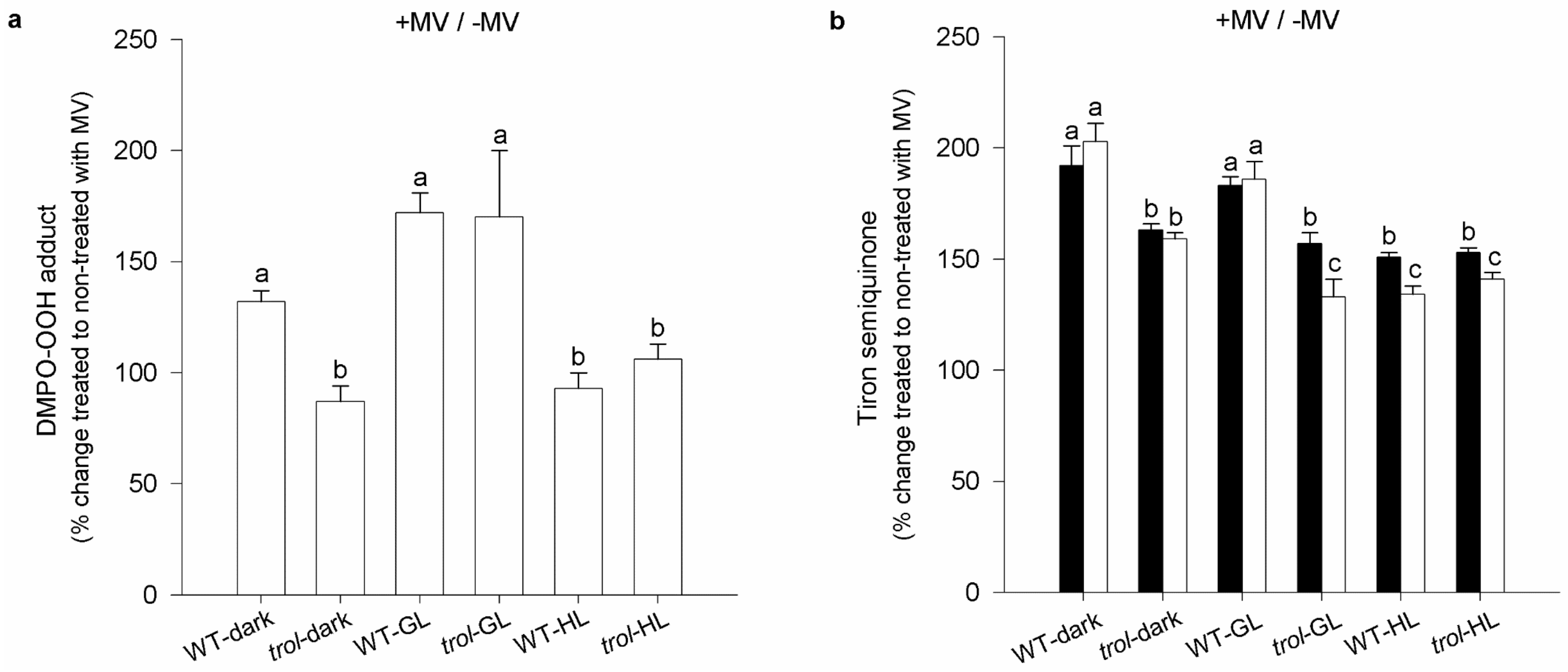

Supplemental Figure S2. The EPR study of DMPO-OOH spin adduct and Tiron semiquinone radical formation in chloroplasts isolated from Arabidopsis *trol* plants acclimated to different light conditions (dark, GL or HL). Chloroplasts were illuminated with photosynthetic light of 100 µmol photons m^{-2} s^{-1} for 30 seconds (white columns) or kept in dark for the equivalent time period (black columns). (a) The superoxide anion production from chloroplasts exposed to MV normalized with respect to the samples not exposed to MV that are taken as a reference for 100% superoxide radical production; (b) Data from chloroplasts exposed to MV normalized with respect to the samples not exposed to MV assumed to represent 100% radical yield. Data (mean ± SE) from three independent measurements were analysed by one-way ANOVA and LSD post-hoc test. Data labelled with the same lower-case letters are not significantly different. Different lower-case letters point to the experimental data which are significantly different ($p<0.05$).

Supplemental Table S2. Experimental EPR data showing free radical production in plants in the presence of DMPO and Tiron spin traps. Data for both WT and *trol* are shown for all at three different plant growth light conditions: dark (D), growth light (GL) and high light (HL) and two different illumination regimes during EPR experiments: dark or illuminated with photosynthetic light of 100 µmol photons m^{-2} s^{-1} for 30 seconds. Mean values from three independent measurements (data in arbitrary units) and standard errors are shown for each measurement.

Plant growth conditions		D		GL		HL	
	EPR conditions	dark	light	dark	light	dark	light
DMPO	WT	123±25	506±5	139±16	395±4	138±5	343±15
	trol	162±34	419±1	91±14	277±41	135±1	273±15
Tiron	WT	1014±10	1096±10	1167±21	1362±10	1357±5	1700±10
	trol	1249±1	1403±10	1382±31	1664±15	1393±1	1690±20

Supplemental References

R1. Aebi, H. Catalase in vitro. *Methods Enzymol.* **105**, 121-126 (1984).

R2. Giannopolities, C. N. & Ries, S. K. Superoxide dismutases. 1. Occurrence in higher plants. *Plant Physiol.* **59**, 309–314 (1977).

R3. Bitensky, L. [Glutathione: Chemical, Biochemical, and Medical Aspects] *Cell Biochemistry and Function* [Dolphin, D., Poulson, R., Avramovic, O. (eds.)] [139] (Wiley-Interscience, New York, 1989).

R4. Verma, S. & Dubey, R. S. Leads toxicity induces lipid peroxidation and alters the activities of antioxidant enzymes in growing rice plants. *Plant Sci.* **164**, 645-655 (2003).

R5. Mukherjee, S. P. & Choudhuri, M. A. Implications of water stress induced changes in the levels of endogenous ascorbic-acid and hydrogen-peroxide in Vigna seedlings. *Physiol. Plant.* **58**, 166–170 (1983).

R6. Levine, R. L., Williams, J. A., Stadtman, E. R. & Shacter, E. Carbonyl assays for determination of oxidatively modified proteins. *Methods Enzymol*. **233**, 346-357 (1994).
